# Necroptosis DAMPens anti-tumor immunity

**DOI:** 10.1038/cddiscovery.2016.33

**Published:** 2016-05-30

**Authors:** K Shibata, Z Omahdi, S Yamasaki

**Affiliations:** 1 Division of Molecular Immunology, Medical Institute of Bioregulation, Kyushu University, Fukuoka, Japan

Pancreatic ductal adenocarcinoma (PDA) causes a highly lethal cancer and is predicted to become the second most common cause of cancer-related death by 2030.^1^ In 2012, the number of deaths due to pancreatic cancer was estimated to have reached 330 000 worldwide. In the latest issue of Nature (6 April 2016), Seifert *et al.* ^2^ demonstrates that necroptosis induces PDA progression through the inhibition of anti-tumor immunity (Figure 1).

From a histological point of view, the most common type of PDA is pancreatic intraepithelial neoplasia (PanIN) which is further classified into three stages, PanIN1-3, based on their severity. KRAS is one of oncogenes frequently found in human pancreatic cancers and its expression was detected in PanIN lesions. The G12D mutation is so far the most common activating mutation of KRAS in human, and genetically mutated mice harboring this mutation recapitulated the disease.^2,3^ Therefore, KRAS^*G12D*^ mice are a useful tool for the study of oncogenic processes of PDA development.

Using this model, the authors first found that major necrosome components, receptor-interacting protein (RIP)1 and RIP3, were highly expressed in PDA. The necrosome is a molecular complex formed during necroptosis, which is one of the cell death mechanisms that is thought to be required for elimination of unwanted or damaged cells due to infection or tumor development. Indeed, *in vitro* experiments showed that deletion of RIP3 in pancreatic ductal epithelial cells from KRAS^*G12D*^ mice increased cell proliferation, which may suggest that RIP3 is also required for tumor progression *in vivo*. Surprisingly, however, this was not the case. Tumor growth of RIP3-deficient KRAS^*G12D*^ mice was attenuated as compared with those in RIP3-sufficient KRAS^*G12D*^ mice. This was also supported by the fact that the treatment of KRAS^*G12D*^ mice with the necroptosis inhibitor, Necrostatin 1s, showed a similar trend. These results led the author to investigate the mechanism by which necrosome-mediated signaling regulates anti-tumor effects against oncogenic progression of PDA.

A key feature of necroptosis is the permeabilization of plasma membranes to release damage-associated molecular patterns (DAMPs), which can trigger robust immune responses and inflammation.^4^ In general, inflammation has a crucial role in cancer development, progression and metastasis. Indeed, a clear linkage between chronic pancreatitis and risk of pancreatic cancer development was reported in both human and mouse.^5,6^ This raised the possibility that tumor microenvironment during PDA development can be regulated by a RIP3-dependent mechanism.

Tumor progression is counter-balanced by various cell populations in the local microenvironment. Myeloid-derived suppressor cells (MDSC), ‘M2-type’ tumor-associated macrophages (TAM) and regulatory T cells are proposed to promote tumor development mainly via inhibition of T cells with anti-tumor activity, whereas tumor-infiltrating IFN-*γ*-producing *αβ* T cells blocked the development of PDA.^7^ RIP3 deficiency in KRAS^*G12D*^ mice reduced frequencies of suppressive immune cells, whereas peritumoral T cells significantly increased. These results indicated that blockade of RIP3-mediated signaling changes the tumor microenvironment to unlock immune suppression status and results in tumor regression.

Next, the authors explored how RIP3-mediated signaling antagonizes immunosuppressive environment. Among the soluble factors tested, CXCL1 was highly expressed in PDA in a RIP3-dependent manner. To evaluate the role of CXCL1, the authors established mice implanted with tumor cells. Anti-CXCL1 treatment in tumor-implanted mice reduced MDSC and TAM infiltration leading to tumor regression. However, frequencies of peritumoral T cells were not increased by treatment with anti-CXCL1 mAb, suggesting other mechanisms downstream of RIP3-mediated pathway.

Mincle is an ITAM-coupled activating C-type lectin receptor, which is induced upon various stimuli and stresses on myeloid cells and recognizes dead cells.^8^ More recently, Mincle was shown to be involved in obesity-induced adipose tissue fibrosis presumably via the recognition of endogenous ligand(s).^9^ The authors detected Mincle expression in PDA-infiltrating myeloid cells. They therefore analyzed the role of Mincle-mediated signaling in PDA progression. To this end, they employed trehalose-6, 6ʹ-dibehenate (TDB), a synthetic glycolipid ligand for Mincle.^10^ Although the administration method of this insoluble lipid was not specified, the authors reported that *in vivo* injection of TDB promoted PDA progression, which is a striking contrast to the recent clinical studies demonstrating its adjuvant effects.^11^ In line with their hypothesis, Mincle deficiency resulted in reduced tumor size and is likely to improve survival compared with Mincle-sufficient condition. In Mincle-deficient tumor microenvironment, high frequencies of IFN-*γ*-producing *αβ* T cells were observed. In contrast, tumor-promoting immunosupressive *αβ* T cells which the authors characterized by the production of IL-10 were decreased. Deletion of Mincle in KRAS^*G12D*^ mice did not affect CXCL1 expression implying that it is regulated independently of Mincle. Collectively, the authors proposed that Mincle pathway may support tumor progression in PDA independently of CXCL1.

Finally, to clarify the importance of T cells in anti-tumor effects, T cells were depleted with anti-CD90.1 mAb in tumor-implanted RIP3-deficient or Mincle-deficient mice. The protective effect mediated by RIP3-Mincle axis was dependent on T cells, but not F4/80+ macrophages.

As mentioned above, patients with PDA have particularly poor prognosis after chemotherapy which targets certain oncogenic pathways. This poor prognosis can be explained by the genetic heterogeneity of metastases present in PDA.^12^ Theoretically, after the somatic recombination processes, T cells can express as many as 10^15^ different receptors and this vast diversity of T cells could meet the requirement to treat genetically heterogeneous PDA. In the tumor microenvironment, T cells with anti-tumor activity are functionally suppressed after receiving inhibitory signals through CTLA4 and PD1. For this reason, T-cell-based immunotherapy targeting CTLA4 and PD1 has been tested to unleash anti-tumor T-cell responses to various tumors. Unfortunately, preclinical studies in PDA patients have been unsuccessful so far because neither anti-CTLA4 nor anti-PD1 mAb treatments showed beneficial effects for survival.^7^ It suggests that an upstream molecule regulating anti-tumor T-cell responses could be considered as the clinical target. In this sense, Mincle blockade by antibodies or small compounds may be a novel therapeutic approach for PDA. Moreover, given that combined blockade of pathways through CXCL1 and Mincle reduced tumor growth more effectively than those in Mincle blockade alone, a therapy aimed at both CXCL1 and Mincle could be the alternative way.

The present study thus shed a new light on clinical targets for the treatment of PDA whereas, at the same time, it raises unsolved questions. First, what is the molecular mechanism underlying the inhibitory signaling of the RIP3-Mincle axis proposed in this study? More specifically, what are the functional endogenous ligands for Mincle in the tumor microenvironment? As mentioned above, the importance of Mincle-mediated signals for host defense against infection implies the caveat of side effects after blocking the signals in immunocompromized PDA patients. It is therefore important to define which pathways orchestrate positive and negative signals via Mincle. Second, which T-cell subsets are responsible for anti-tumor effects against PDA? Identification of antigens recognized by anti-tumor T-cell subsets could help establish successful T-cell-based immunotherapy. Based on this study, DAMPening of PDA may therefore be a promising approach but efforts are still underway.

## Figures and Tables

**Figure 1:**
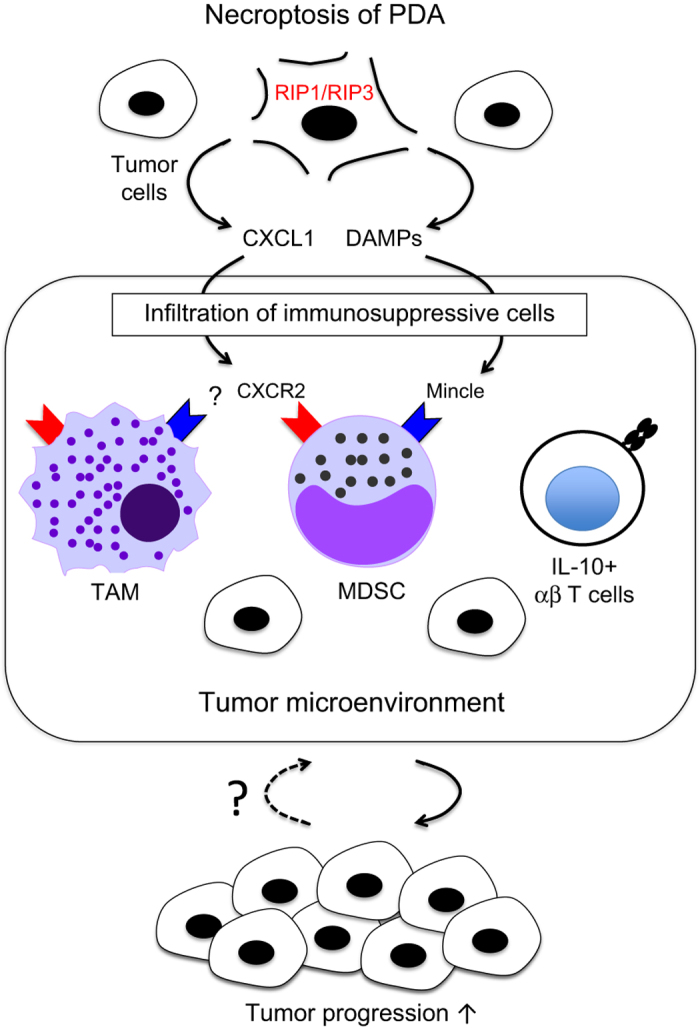
RIP1/RIP3-dependent necroptosis promotes pancreatic tumor progression. In PDA, RIP1/RIP3-dependent necroptosis induces the upregulation of CXCL1 which promotes the recruitment of immunosuppressive cells including MDSCs, TAM and IL-10-producing *αβ* T cells. In addition, the recognition of endogenous ligands through the receptor Mincle and possibly other unidentified receptors is also involved in this process independently of CXCL1.

## References

[bib1] Rahib L et al. Cancer Res 2014; 74: 2913–2921. 24840647 10.1158/0008-5472.CAN-14-0155

[bib2] Seifert L et al. Nature 2016; 532: 245–249. 27049944 10.1038/nature17403 PMC4833566

[bib3] Biankin AV et al. Nature 2012; 491: 399–405. 23103869 10.1038/nature11547 PMC3530898

[bib4] Kaczmarek A et al. Immunity 2013; 38: 209–223. 23438821 10.1016/j.immuni.2013.02.003

[bib5] Malka D et al. Gut 2002; 51: 849–852. 12427788 10.1136/gut.51.6.849 PMC1773474

[bib6] Guerra C et al. Cancer Cell 2007; 11: 291–302. 17349585 10.1016/j.ccr.2007.01.012

[bib7] Ying H et al. Genes Dev 2016; 30: 355–385. 26883357 10.1101/gad.275776.115 PMC4762423

[bib8] Yamasaki S et al. Nat Immunol 2008; 9: 1179–1188. 18776906 10.1038/ni.1651

[bib9] Tanaka M et al. Nat Commun 2014; 5: 4982. 25236782 10.1038/ncomms5982

[bib10] Ishikawa E et al. J Exp Med 2009; 206: 2879–2888. 20008526 10.1084/jem.20091750 PMC2806462

[bib11] Hansen J et al. Cancer Immunol Immunother 2012; 61: 893–903. 22095092 10.1007/s00262-011-1156-6 PMC11028613

[bib12] Yachida S et al. Nature 2010; 467: 1114–1117. 20981102 10.1038/nature09515 PMC3148940

